# KSHV ORF57, a Protein of Many Faces

**DOI:** 10.3390/v7020604

**Published:** 2015-02-10

**Authors:** Vladimir Majerciak, Zhi-Ming Zheng

**Affiliations:** Tumor Virus RNA Biology Section, Gene Regulation and Chromosome Biology Laboratory, National Cancer Institute, National Institutes of Health, 1050 Boyles Street, Frederick, MD 21702, USA; E-Mail: Vladimir.Majerciak@nih.gov

**Keywords:** KSHV, ORF57 protein, viral expression, posttranscriptional regulator, RNA processing

## Abstract

Kaposi’s sarcoma-associated herpesvirus (KSHV) ORF57 protein (also known as mRNA transcript accumulation (Mta)) is a potent posttranscriptional regulator essential for the efficient expression of KSHV lytic genes and productive KSHV replication. ORF57 possesses numerous activities that promote the expression of viral genes, including the three major functions of enhancement of RNA stability, promotion of RNA splicing, and stimulation of protein translation. The multifunctional nature of ORF57 is driven by its ability to interact with an array of cellular cofactors. These interactions are required for the formation of ORF57-containing ribonucleoprotein complexes at specific binding sites in the target transcripts, referred as Mta-responsive elements (MREs). Understanding of the ORF57 protein conformation has led to the identification of two structurally-distinct domains within the ORF57 polypeptide: an unstructured intrinsically disordered *N*-terminal domain and a structured α-helix-rich *C*-terminal domain. The distinct structures of the domains serve as the foundation for their unique binding affinities: the *N*-terminal domain mediates ORF57 interactions with cellular cofactors and target RNAs, and the *C*-terminal domain mediates ORF57 homodimerization. In addition, each domain has been found to contribute to the stability of ORF57 protein in infected cells by counteracting caspase- and proteasome-mediated degradation pathways. Together, these new findings provide insight into the function and biological properties of ORF57 in the KSHV life cycle and pathogenesis.

## 1. Introduction

Productive viral replication depends on the efficient and coordinated expression of viral genes. Kaposi’s sarcoma-associated herpesvirus (KSHV) encodes more than 100 viral genes, which are expressed in a time-dependent manner [1,2,3,4], whereas only few KSHV genes are expressed during the viral latency stage, cascaded expression of all KSHV genes occurs during viral lytic infection. The virus-encoded replication and transcription activator (Rta or ORF50) is essential and sufficient to initiate the KSHV lytic cycle [5,6,7], but completion of the productive KSHV lytic cycle also requires expression of the ORF57 protein. Deletion of ORF57 from the virus genome leads to the inefficient expression of viral lytic genes and abortive viral replication [8,9,10]. Therefore, ORF57 acts on gene expression after ORF50 initiates transcription. This function of ORF57 is responsible for fulfilling the KSHV lytic cycle and viral replication.

KSHV ORF57 is a nuclear protein composed of 455 amino acid (aa) residues. Functional homologues of ORF57 have been found within the entire family of herpesviruses, of which the ICP27 protein of herpes simplex virus type 1 (HSV-1), IE4 of varicella-zoster virus (VZV), UL69 of human cytomegalovirus (HCMV), EB2 of Epstein-Barr virus (EBV), and ORF57 of herpesvirus saimiri (HVS) are well characterized [11,12,13,14]. Similarly to KSHV ORF57, all homologues regulate viral gene expression at the posttranscriptional level by interacting with cellular RNA-binding proteins. However, KSHV ORF57 often deviates from its homologues in several aspects of RNA processing.

## 2. The Primary Structure of ORF57 Protein

**Figure 1:**
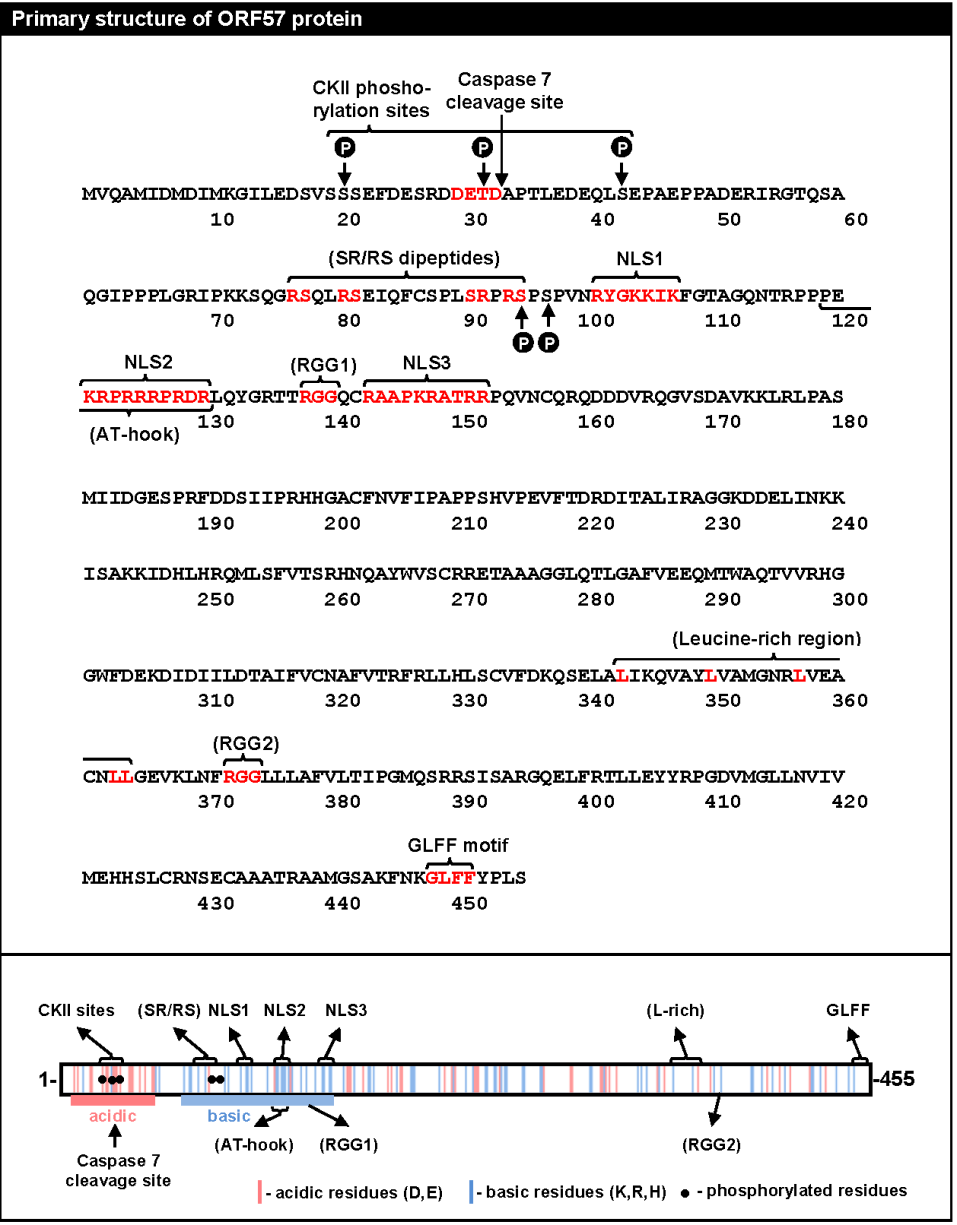
Protein sequence and putative motifs of ORF57. (Top) The amino acid sequence of ORF57 with putative motifs indicated with brackets and the residues making up the motifs shown in red. Phosphorylation sites (P in black circle) are also indicated. (Bottom) A summary of the primary structure of the ORF57 protein (aa 1–455) with the distribution of acidic (red lines, D: aspartic acid, E: glutamic acid) and basic (blue lines, K: lysine, R: arginine, H: histidine) amino acids. The linear motifs identified based on the analysis of the ORF57 aa sequence are shown above and below (CKII-casein kinase II, SR/RS-serine/arginine and arginine/serine dipeptides, NLS-nuclear localization signal, L-rich-leucine-rich domain, GLFF motif-glycine/leucine/phenylalanine/phenylalanine motif, AT hook-adenine-thymine hook, RGG-arginine/glycine/glycine). The functionally unconfirmed putative motifs are in parentheses. The filled circles represent the mapped ORF57 phosphorylation sites.

KSHV ORF57 represents a unique viral protein with no significant sequence homology to any known cellular proteins. It does share limited homology with its homologues in the herpesvirus family, but this only amounts to about 30% sequence homology to even its evolutionarily closest homologues EBV EB2 and HVS ORF57 [15]. An initial motif analysis of the KSHV ORF57 aa sequence has identified several sequence motifs remotely resembling those found in cellular RNA-binding proteins (Figure 1). These include two simple RGG motifs (RGG1, aa 138–140, and RGG2, aa 372–374), which are similar to the RGG-box of RNA-binding proteins; four serine/arginine or arginine/serine dipeptides (SR/RS, aa 77–95), which are common in cellular SR proteins; a nonconsensus putative AT (adenine-thymine) hook (aa 119–130), a motif typical of DNA binding proteins; a putative leucine-rich region (L-rich, aa 343–364) resembling the leucine zipper of cellular transcription factors; and a γ-herpesvirus-specific “GLFF” motif (aa 448–451) of unknown function. In addition to these motifs, the *N*-terminal half of ORF57 is enriched in polar residues to form a short acidic region (~aa 7–52), followed by a basic region with a high content of arginine residues (aa 69–152) that harbors all three functionally redundant nuclear localization signals (NLSs; NLS1, aa 101–107; NLS2, aa 121–130; NLS3, aa 143–152) (Figure 1) [16].

Despite some sequence resemblance to known motifs, most putative motifs identified in ORF57 have not been characterized, and their authentic nature remains in question. In general, most putative motifs in the ORF57 protein are highly degenerated from the consensus aa sequences found in the cellular counterparts and are often missing residues essential for their function. For example, the described AT hook lacks a core “PRGRP” sequence, and functional RGG boxes require at least 2 or 3 repeats of an RGG or RG motif separated by 0–4 residues to mediate RNA binding [17,18]. The predicted motifs among the homologues in the herpesvirus family are highly variable, both in aa composition and in physical position, even among the closest ORF57 homologues. This structural variability appears to contradict the observed conservation of function among some of the homologues [9,19,20,21]. Thus, these putative motifs can be simply viewed as the rudiments of functional motifs degenerated over million years of evolution or, in some cases, just as a coincidental cluster of amino acid residues. To avoid confusion in future descriptions of ORF57, these motifs should be omitted or labeled as “putative” motifs unless their functionality is clearly validated by experimental approaches.

## 3. Expression and Localization of ORF57 Protein

Early in KSHV lytic infection, ORF57 is expressed as an abundant monocistronic RNA [15,22]. ORF57 transcription is transactivated when Rta binds to the ORF57 promoter, which contains multiple Rta-responsive elements (RREs), within a complex containing cellular cofactors such as RBP-Jκ (recombination signal binding protein for immunoglobulin kappa J region) [23,24,25]. The transcript is polyadenylated by using a polyadenylation signal downstream of the ORF57 open reading frame (ORF) region. The same polyadenylation signal is also used for the polyadenylation of a bicistronic ORF56-ORF57 RNA transcriptionally initiated from a promoter upstream of the colinear ORF56 gene [2,22]. The primary ORF57 transcript or pre-mRNA has two exons divided by a small suboptimal intron that is efficiently spliced to form an ORF57 mRNA [22].

In experimental systems, a second intron has been detected. For example, a second intron was noticed in the 3' end of ORF57 RNA in iSLK-219 cells, a stable cell line harboring a recombinant KSHV.219 virus [4], and could be spliced at low frequency to generate a double-spliced mRNA encoding a truncated ORF57 protein with the first 266 aa of ORF57 plus an additional 33 aa from a new out-of-frame exon 3. This splicing is most likely from the usage of a cryptic splice site in the recombinant KSHV.219 virus due to the insertion of a reporter cassette containing green and red fluorescent proteins (GFP and RFP) downstream of ORF57 [26] and occurs only in cells infected with the recombinant KSHV.219 virus, not in the cells lytically infected with a native KSHV genome [4]. Thus, further analysis is needed to confirm this result.

The expression of KSHV ORF57 is also regulated by cellular RNA export factors and cofactors at the posttranscriptional level. RNA export factors UAP56 and URH49, and RNA export cofactors RBM15 and OTT3, modulate KSHV ORF57 expression. Knockdown of each factor with RNAi (RNA interference) decreases ORF57 expression. The reduced expression of these factors causes a deficiency in nuclear export of ORF57 RNA and consequently decreased expression of ORF57 and its targets in the context of the KSHV genome [27].

ORF57 protein exhibits predominantly nuclear localization and its concentration is increased in SRSF2 (SC35)-positive nuclear speckles that are also enriched in other splicing factors [16,20]. The nuclear localization of ORF57 is governed by three NLSs clustered in the ORF57 *N*-terminus [16] (Figure 1). Although the multiple NLSs exist in ORF57 homologues, they share only limited sequence and position conservation [28,29]. ORF57 NLSs exhibit functional redundancy, with each one being sufficient for ORF57 nuclear localization [16]. However, the simultaneous mutation of any two of the three NLSs eliminates ORF57 activities, indicating that the NLS sequences play a role beyond the nuclear localization of ORF57 protein [16]. ORF57 is not a nucleolar protein [16,20,30]. In KSHV-infected PEL (primary effusion lymphoma) cells, ORF57 protein is rarely seen in the nucleolus [20,30,31] and thus the published study showing ORF57 in BCBL-1 nucleoli [32] is most likely an artifact. ORF57 shuttles between the nucleus and the cytoplasm, but a functional nuclear export signal has not yet been defined.

## 4. Regulation of KSHV Gene Expression by ORF57

### 4.1. ORF57 Does Not Directly Export Viral RNAs

In general, the efficient nucleocytoplasmic export of mammalian RNA transcripts is coupled to RNA splicing through exon-junction complexes (EJC). Inhibition of RNA splicing impairs the export of unspliced, intron-containing RNAs from the nucleus to the cytoplasm. In contrast to the majority of mammalian pre-mRNA transcripts, which contain introns, approximately two-thirds of KSHV gene transcripts are intronless. They are therefore expected to be exported from the nucleus to the cytoplasm inefficiently, and to therefore be poorly expressed. However, the KSHV genome robustly expresses these intronless genes during lytic infection for viral multiplication and pathogenesis. KSHV ORF57 protein was initially thought to be responsible for this robust expression of viral intronless genes in infected cells because its homologues in other members of the herpesvirus family promote the export of viral intronless transcripts [29,33,34,35].

The involvement of ORF57 in RNA export was first tested using a pMD138-derived pCMV128 system expressing a chimeric RNA containing a chloramphenicol acetylase (CAT) ORF as a reporter within its suboptimal, inefficiently spliced intron. Because CAT expression requires the RNA export of the unspliced RNA, the observed increase in CAT activity in the presence of ORF57 expression was interpreted as promotion of RNA export by ORF57 [36]. However, this result can also be attributed to ORF57-mediated enhancement of RNA stability, which has been recognized lately as a major function of ORF57 [16,30,37,38,39]. In fact, a recent report indicated that direct tethering of ORF57 to the CAT-reporter RNA does not increase the export of the unspliced RNA or CAT expression [40].

ORF57 interacts with Aly/REF, a cellular cofactor originally considered to be essential for RNA export [36], as do other homologous proteins in the herpesvirus family [29,33,41]. Aly/REF is an RNA export adaptor that interacts with RNA and TAP/NXF1, an mRNA export receptor, and is a component of the TREX (transcription and export) complex that links the majority of spliced and unspliced RNAs to the cellular export machinery [42,43] through its interaction with the RNA 5' cap-binding protein CBP80 [44]. Thus, the simultaneous interactions of ORF57 with viral transcripts and Aly/REF would allow viral RNAs to access the nuclear export machinery [45]. However, this presumption lacks direct supporting evidence. The ability of ORF57 to enhance the expression of viral intronless RNAs appears not to be affected initially by knockdown of Aly/REF with siRNAs [16] and later by disruption of the ORF57–Aly/REF interaction by mutation of the Aly/REF binding site in ORF57 [30]. An ORF57 mutant incapable of binding Aly/REF, however, did rescue the replication of an ORF57-null virus and moderately increased the expression of ORF57 targets [21]. These studies show that the effect of ORF57 to accumulate viral intronless RNAs is independent of Aly/REF. Other studies also indicate that Aly/REF is dispensable for metazoan RNA export in general [46,47] and for the export function of the HSV ICP27 protein [48]. In Aly/REF-depleted cells, ORF57 activity was later explained by ORF57 interaction with the UIF protein, which interacts with UAP56, a component of TREX whose function is redundant with that of Aly/REF [49,50]. Again, how this interaction of ORF57 with UIF contributes to ORF57 function remains largely unknown. Experimental data show that an ORF57 mutant deficient in Aly/REF binding also failed to interact with UIF [50], but the same mutant in another study was capable of causing viral RNAs to accumulate [21]. Consistent with this, depletion of either Aly/REF or UIF in cells produced little effect on ORF57 function for the RNAs tested [50]. Although depletion of both Aly/REF and UIF in the ORF57-transfected cells did reduce the accumulation of ORF47 mRNA in the cytoplasm [50], this double depletion might also affect the expression of ORF57 protein and thus its RNA accumulation function of ORF47 because RNA export factors and cofactors are required for the expression of ORF57 [27].

**Figure 2:**
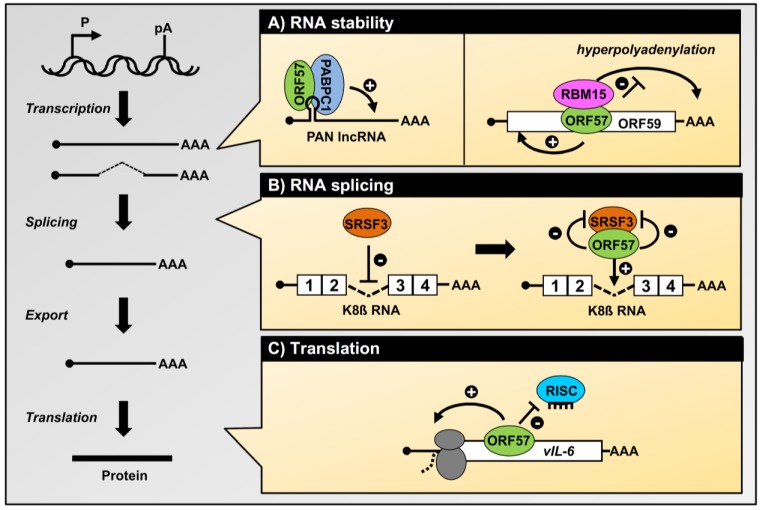
Roles of ORF57 in the posttranscriptional processing of KSHV transcripts. The diagram on the left depicts the major steps of RNA biogenesis, including transcription, RNA splicing, RNA nucleocytoplasmic export, and RNA translation into protein. (**A**) Enhancement of the stability of KSHV intronless RNAs by ORF57. Cooperative binding of ORF57 with PABPC1 to an ORF57/Mta-responsive element (MRE, hairpin) protects the KSHV PAN lncRNA from degradation in the nucleus [37,56]. Similarly, ORF57 binding to KSHV ORF59 RNA enhances ORF59 RNA stability by preventing its hyperpolyadenylation by RBM15 protein [38]; (**B**) ORF57 promotes K8β RNA splicing by attenuating the suppressive activity of splicing factor SRSF3 (SRp20). ORF57 interacts with SRSF3 and blocks SRSF3 interaction with an intronic region of K8β RNA [31]; (**C**) The role of ORF57 in protein translation. ORF57 inhibits miRNA-induced translation inhibition by displacing RISC (RNA-induced silencing complex) from vIL-6 (viral interleukin-6) via competitive binding to the ORF57 MRE, which contains a miR-1293 binding site [58].

Cytoplasmic accumulation of the targeted RNAs could be a result of ORF57-mediated RNA stability, rather than ORF57-mediated export, initially proposed by our group [16] and later Swaminathan’s group [30] when Aly/REF was found being not essential for ORF57-RNA interaction [16] and its function [16,30]. Subsequently, the ORF57–Aly/REF interaction promotes the stability of KSHV nuclear polyadenylated (PAN) RNA, rather than its export, which was further confirmed by Conrad’s group [51]. PAN RNA is a KSHV-specific, intronless long non-coding RNA (lncRNA) involved in virus reactivation and modulation of host immune response [52,53,54]. PAN RNA is highly expressed during viral lytic infection; with ~25,000 copies per cell, it accounts for over 80% of the viral transcriptome. ORF57 enhances the expression of PAN RNA [8,55] by directly interacting with it [37,39,56,57]. The finding that PAN is mainly retained in the nucleus in the presence of ORF57 contradicts the proposed RNA export function of ORF57 [45]. ORF57 also enhances overall RNA levels of viral intronless coding RNAs (KSHV ORF59 and ORF47) without affecting their nucleocytoplasmic ratio, as shown with Northern blots of fractionated RNA or directly in FISH experiments [21,30,38,40]. Thus, the observed increase of the cytoplasmic levels of these RNAs results from an overall increase in RNA stability mediated by ORF57 (Figure 2).

### 4.2. Stabilization of Viral Intronless Transcripts by ORF57

RNA stability is one of the fundamental mechanisms in the regulation of gene expression that determines the final RNA concentration at the posttranscriptional level [59,60,61]. The role of ORF57 in PAN RNA stability as described above is one of the best-documented examples (Figure 2). Lack of RNA splicing, export, and translation in the biogenesis of PAN RNA enables us to illustrate ORF57’s effect on RNA stability. That PAN RNA responds to ORF57 for its robust expression has been known for more than a decade [55], but the mechanism of how ORF57 promotes PAN RNA expression became understood only recently. Using an anti-ORF57 UV cross-linking immunoprecipitation (CLIP) technique for KSHV natively infected B cells with KSHV lytic infection, we demonstrated that ORF57 protein intimately binds to PAN RNA in JSC-1 B cells and generates an intermolecular cross-link in the cells under UV irradiation [58]. In combination with RNase and proteinase digestion and cDNA clone screening of ORF57-protected RNA fragments, we identified a MRE at the 5' PAN binding to ORF57 protein, leading to a significant (3-fold) increase in PAN half-life [37]. Other studies have shown similar results by transiently expressing ORF57 and PAN [39,57]. However, the caveat in the later study is that the ORF57 showed only increasing the half-life of an ENE (expression and nuclear retention element)-lacking mutant PAN, but not that of the ENE-containing wild-type PAN [39]. The ENE previously identified at the PAN 3' end [62,63] appears not to be essential for ORF57 to stabilize PAN RNA and can be deleted without affecting ORF57 function in accumulation of PAN expression [37,39,57]. Thus, the biological significance of the ENE for PAN RNA expression during KSHV infection remains to be investigated. Further studies show that the identified 5' PAN MRE forms a secondary RNA structure with three stem-loops (MRE I-III) and contains a 9-nt core in the loop of MRE II for the binding of ORF57 [37,57] and host PABPC1 (polyA binding protein, cytoplasmic 1) [37]. In the context of PAN RNA, the PAN MRE is highly active in response to ORF57, but only partially active in the context of heterologous viral and non-viral transcripts [37,57].

Binding of PABPC1 to the 9-nt core of PAN MRE is important for ORF57 interactions with PAN [56]. However, ORF57 and PABPC1 have opposing functions in modulating PAN steady-state accumulation. In the absence of ORF57, PABPC1 suppresses PAN accumulation. In the presence of ORF57, the *N*-terminus of ORF57 interacts with the RNA-recognition motif (RRM) of PAPBC1 and alleviates the negative effect of PAPBC1 on PAN [56]. ORF57, whether expressed exogenously or during KSHV infection, induces the translocation of PABPC1 from the cytoplasm to the nucleus [56], similar to the findings on nuclear translocation of cytoplasmic PABPC1 by viral SOX (ORF37) protein [64] and colocalizing PABPC1 with nuclear PAN RNA during lytic KSHV infection [65].

ORF57 enhances the stability of protein-encoding ORF59 RNA by increasing its half-life in the cells [38]. ORF57 binds ORF59 RNA in JSC-1 cells with lytic KSHV infection was initially discovered by *in vivo* UV-crosslinking and anti-ORF57 CLIP in 2006 [16]. ORF59 RNA accumulation in cells is also affected by the expression of RBM15 and OTT3, two members of the SPEN protein family that interact with ORF57 [38]. Although RBM15 does not promote ORF59 RNA stability, ectopic expression of RBM15 leads to nuclear accumulation and hyperpolyadenylation of nuclear-retained ORF59 RNA. Co-expression of ORF57 prevents RBM15-mediated hyperpolyadenylation and nuclear retention of ORF59 RNA and releases ORF59 RNA from the RBM15 complexes [38], thereby enhancing ORF59 stability (Figure 2). A functional MRE that mediates ORF59 sensitivity to ORF57 regulation has been mapped to the 5' ORF59 RNA [58,66]. ORF57 specifically binds to a stem-loop region from nt 96596-96572 of the MRE and internal deletion of the MRE from ORF59 leads to poor export, but accumulation of nuclear ORF59 RNA in the presence of ORF57 or RBM15. ORF57 also increases the state-steady levels of several other viral RNAs, including ORF56 (viral primase), ORF47 (glycoprotein L), and viral interleukin 6 (vIL-6) [22,40,58]. However, further studies are needed to elucidate the underlying mechanisms by which ORF57 participates in their enhanced expression.

Multiple pathways have been identified to regulate RNA stability at all stages of RNA biogenesis, both in the nucleus and in the cytoplasm [67]. To date, it remains unclear which pathway is directly targeted by ORF57. The finding that ORF57 stabilizes nuclear PAN RNA and the predominantly nuclear ORF47 RNA suggests that ORF57 acts in the nucleus, but does not rule out the possibility that ORF57 may target multiple RNA degradation pathways.

### 4.3. ORF57 Functions As a Viral Splicing Factor

Based on the characteristics of HSV-1 ICP27, ORF57 was originally proposed to inhibit RNA splicing. However, the KSHV genome encodes at least one-third of its genes with one or more introns that require RNA splicing for their expression and productive infection [68]. It seems unlikely that a virus would encode a protein that prevents its own RNA splicing and blocks the expression of its own genes. In fact, knocking out the ORF57 gene in the KSHV genome results in the accumulation of several unspliced viral pre-mRNAs, including those for the KSHV ORF50 (Rta) and K8 (k-bZIP) RNAs [20]. In cotransfection assays, ORF57 promotes RNA splicing of these transcripts in the absence of other viral factors [20]. It has been noted that ORF57 mainly promotes RNA splicing of pre-mRNAs containing suboptimal introns, not RNAs having optimal introns [20].

Although ORF57’s ability to promote RNA splicing is independent of other viral factors, it requires the interaction of ORF57 with several cellular splicing factors (SRSF1, SRSF3, *etc.*) and other components of the spliceosome, including SM proteins and U snRNPs (uridine-rich small nuclear ribonucleoproteins) [20]. Together with the finding that ORF57 associates selectively with unspliced pre-mRNAs, but not with fully spliced mRNA, in a splicing reaction, these data indicates that ORF57 is recruited into the spliceosome during spliceosome assembly and affects splice site selection [20]. ORF57 attenuation of the suppressive activity of SRSF3 (formerly SRp20) has recently been recognized as the molecular mechanism behind ORF57-mediated splicing of KSHV K8β RNA [31]. SRSF3, the smallest member of the human SR protein family and a proto-oncoprotein [69], binds to multiple regions of a suboptimal K8β intron and suppresses its splicing. ORF57 directly interacts with the RRM domain of SRSF3, thereby preventing SRSF3 from binding to a putative branch point region of the K8β intron and increasing K8β splicing (Figure 2). In addition to K8β, ORF57 promotes RNA splicing of several other pre-mRNAs negatively regulated by SRSF3. ORF57 homologues in the herpesvirus family also interact with SRSF3, but these interactions appear to produce different consequences. HSV-1 ICP27 interacts with SRSF3 for inhibition of RNA splicing and for overall RNA export efficiency during HSV-1 infection, whereas EBV EB2 binds SRSF3 to modulate alternative RNA splicing of cellular STAT1 transcripts [70,71,72,73].

### 4.4. ORF57 Promotes Protein Translation

Despite its predominantly nuclear localization, a small fraction of ORF57 protein remains in the cytoplasm, where ORF57 associates with the translating ribosomes and cellular factors related to protein translation. These observations suggest that ORF57 might play a role in protein translation. ORF57 directly interacts with PCBP1 (poly(rC)-binding protein 1), a multifunctional cellular RNA-binding protein involved in activating protein translation from an internal ribosome entry site (IRES) [74], and promotes protein translation from various IRES elements independently or in cooperation with PCBP1. How ORF57 promotes IRES-mediated translation remains unknown. Because both PCPB1 and ORF57 affect RNA stability, the functional effects of ORF57 and PCBP1 interaction on translation should be re-evaluated.

ORF57 interacts with PYM protein [75], a cellular scaffold protein. In the nucleus, PYM binds the Y14-Mago heterodimer moiety of the EJC core that is recruited to RNA during RNA splicing, and in the cytoplasm, it binds the 40S ribosomal subunit that associates with the translation initiation complex [76]. As a result, PYM stimulates a pioneer round of translation of spliced mRNA transcripts and disassembles the EJC from mRNAs in the cytoplasm after the pioneer round of translation [77,78]. Thus, direct interactions between PYM and ORF57 [75] link ORF57 with the components of the translation initiation complex, presumably to promote translation of several KSHV intronless transcripts. However, the mechanism by which ORF57 acts within the translation initiation complex to affect the translation initiation of the KSHV intronless RNAs is worth exploring further.

By using an anti-ORF57 CLIP assay to search for genome-wide RNA targets of ORF57 in JSC-1 B cells, we recently identified 11 viral transcripts as ORF57 targets and found that the coding region of vIL-6 contains an MRE composed of two motifs for ORF57 binding (MRE-A and MRE-B). ORF57 binds MRE-A to regulate vIL-6 RNA stability. MRE-B has a binding site for cellular miR-1293 [58]. Mutation of the miR-1293 binding site in vIL-6 RNA or blocking miR-1293 activity enhances the translation of vIL-6. ORF57 binds MRE-B, preventing the miR-1293–Ago2 RISC from associating with vIL-6 RNA, and thereby enhancing the translation of vIL-6 [58] (Figure 2). In fact, the abundance of miR-1293 and vIL-6 protein expression is negatively correlated in KSHV-infected B cells in the lymph nodes of patients with multicentric Castleman disease. In these patients, vIL-6 in the lymph nodes is mainly present in the mantle zone where miR-1293 levels are low, but it is not found in the germinal centers that are rich in miR-1293 [79]. Similar to its effect on vIL-6, ORF57 promotes the expression of human IL-6 (hIL-6) by competing with miR-608 for a binding site in the ORF region that corresponds to the binding region in vIL-6 [79]. Together, these observations provide not only direct evidence for the functionality of miRNA-binding sites within the coding region, but also disclose how ORF57 contributes to high levels of expression of both vIL-6 and hIL-6 during KSHV infection, which are necessary for the growth of KSHV-infected cells via autocrine and paracrine mechanisms [80,81,82].

### 4.5. Other Putative Functions of ORF57

ORF57 was proposed to contribute to transcriptional control partially based on its interaction with KSHV ORF50 (Rta) and k-bZIP (K8) [83,84,85,86], and its homologs in other herpesviruses stimulate transcriptional initiation [87], but this has been controversial. ORF57 was found to activate multiple Rta-dependent promoters independently or in synergy with Rta. In either case, ORF57 activity was highly variable depending on the individual promoters and cell types used in the studies [83,84]. The synergetic effect of ORF57 on Rta-dependent transcription requires an RRE, but the PAN RRE is not sufficient to achieve ORF57-Rta synergy in an HSP70 (70-kDa heat shock protein) promoter [84] and is not essential for transactivation by ORF57. Thus, the biological significance of the direct binding of ORF57 protein to the DNA via a putative, nonconsensus “AT hook” motif [84] remains unknown. How ORF57 affects transcription remains unclear, but could be explained by the posttranscriptional activities of ORF57 in promoting Rta and/or reporter gene expression through stabilization of their RNAs or by its effect on translation. Several lines of evidence speak against the direct involvement of ORF57 in the regulation of transcription. First, an ORF57 mutant containing aa 329–455, which lacks a functional nuclear localization signal [16] and would presumably be a cytoplasmic protein, was able to transactivate an ORF50 promoter [83]. Second, ORF57 transactivation was partially, but not fully resistant to actinomycin D, a transcription elongation inhibitor, indicating a posttranscriptional role for ORF57 [84]. Third, a recent chromatin immunoprecipitation and microarray assay with ORF57 protein showed that ORF57 associates only with K8-interacting promoters as well as oriLyt [85], indicating its indirect recruitment to viral genome DNA via a DNA-binding protein K8. Alternatively, ORF57 might be recruited to the viral genome regions co-transcriptionally via its interactions with other viral transcription factors and cellular splicing factors, similar to the observed interaction of ICP27 with RNA polymerase II [88]. In fact, one of the reported ORF57 DNA binding sites within the PAN locus overlaps with the previously mapped MRE element but not with the PAN promoter [85].

After the discovery of a functional MRE in the 5' PAN region that increases PAN stability, we compared wt PAN and its mutant with point mutations in the 9-nt MRE core for their expression in HEK293 cells either in the context of a CMV promoter or the native PAN promoter [37]. Although the CMV promoter drives a minimal level of PAN expression, the expression level of wt PAN, but not of the mutant PAN, was remarkably increased in the presence of ORF57 [37]. In the context of the native PAN promoter, which depends on Rta transactivation, a low expression level of wt PAN, but not its mutant, was activated by Rta in a dose-dependent manner. Again, the expression of Rta-transactivated wt PAN, but not its mutant, from the native PAN promoter was increased several-fold in the presence of ORF57, suggesting that the mutant PAN lacking the 9-nt MRE core was unstable even when the PAN promoter was highly active. Together, these data clearly indicate that ORF57 does not function to promote transcription regardless of which promoter is present, but rather stabilizes the transcribed PAN RNA through its interaction with the 9-nt MRE core at the posttranscriptional level. The described MRE was also confirmed independently by another group as a core RNA element for ORF57 binding and activity [57].

ORF57 may have a role in the induction of genome instability during KSHV lytic infection. Genome instability is manifested by increased numbers of DNA double-strand breaks due to activation of cellular DNA damage. ORF57 sequesters the TREX complex from the site of transcription and causes a double-strand break response and significant DNA damage. Overexpression of ORF57 results in the formation of RNA:DNA hybrids (R-loops) vulnerable to DNA breaks [89].

## 5. Structural Determinants of ORF57 Function and Stability

### 5.1. ORF57 Secondary and Tertiary Structure

**Figure 3:**
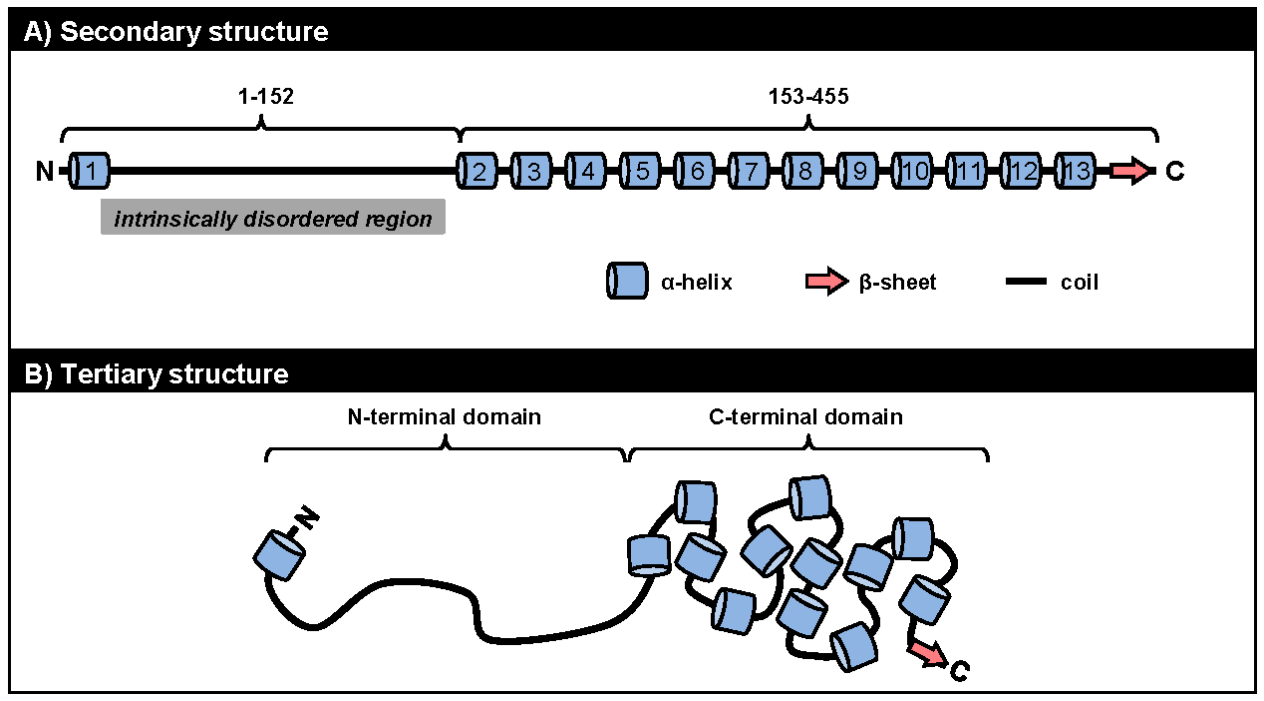
The structural conformation of ORF57 protein. The ORF57 secondary structure (**A**) predicted by the PSIPRED (Psi-blast based secondary structure prediction) version 3.0 prediction tool (http://bioinf.cs.ucl.ac.uk/psipred/) [95] is composed of 13 α-helixes (barrels), one β-sheet (thick arrow), and unstructured coils (thick lines) forming two structurally distinct domains: a unstructured *N*-terminal domain (aa 1–152) with an intrinsically disordered region (IDR) and an α-helix-rich *C*-terminal domain (aa 153–455); The proposed ORF57 tertiary structure (**B**) consists of a flexible linear *N*-terminal domain and a rigid globular *C*-terminal domain [90].

**Figure 4:**
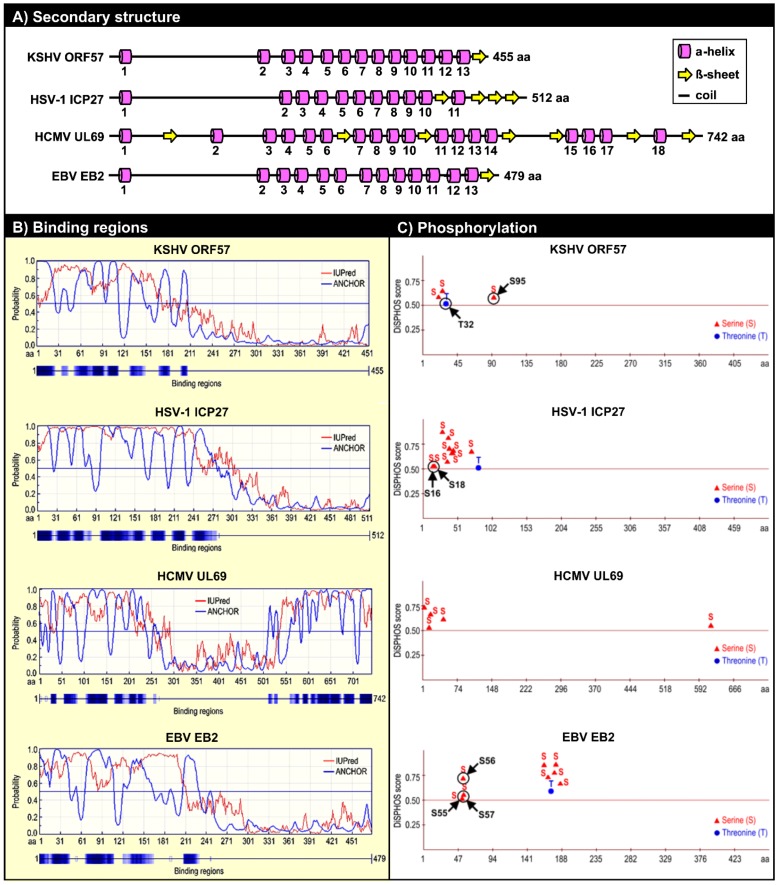
Structural similarities among KSHV ORF57 homologues. (**A**) Conservation of the secondary structure among KSHV ORF57 homologues: HSV-1 ICP27, HCMV UL69 and EBV EB2 based on PSIPRED version 3.0 prediction tool [95]. Numbers under each α-helix represent their relative positions from the *N*- to *C*-terminus. Numbers at the end of each protein represents the protein length in amino acid residues (aa). The drawings are not to scale. This panel is reproduced with permission from [90]; (**B**) Prediction of the disordered binding regions of KSHV ORF57, HSV-1 ICP27, HCMV UL69, and EBV EB2 both by IUPred (http://iupred.enzim.hu/) and by ANCHOR software (http://anchor.enzim.hu/) [96]; (**C**) Phosphorylation sites predicted from KSHV ORF57, HSV-1 ICP27, HCMV UL69, and EBV EB2 proteins by DISPHOS 1.3 software for viruses group predictor, with a threshold value above 0.5 (http://www.dabi.temple.edu/disphos/) [97]. Arrows indicate the experimentally confirmed phosphorylation sites [90,98,99]. S, serine; T, threonine.

We currently know very little about the structures of ORF57 and its homologues. Because ORF57 is poorly expressed in bacteria and is prone to precipitation during purification, NMR and X-ray crystallography cannot be used to study its structural biology. A recent *in silico* analysis to predict the secondary structure of ORF57 demonstrated several fundamental features of the protein conformation: (1) ORF57 exhibits overall low structural complexity, with only one third of all residues being in a secondary structure; (2) ORF57 consists almost exclusively of α-helixes, with only one β-sheet; and (3) the identified structural elements are unevenly distributed along the ORF57 polypeptides, with the majority clustered in the ORF57 *C*-terminal half (Figure 3A) [90]. Using limited proteolysis of both purified and natively expressed ORF57, we confirmed the predicted secondary structure and identified two structurally distinct domains in ORF57: an unstructured *N*-terminal domain (aa 1–152) and an α-helix-rich *C*-terminal domain (aa 153–455). The intrinsically disordered region (IDR) in the unstructured ORF57 *N*-terminus encompasses both acidic and basic regions (Figure 3 and Figure 3A). Besides lacking structure, the ORF57 IDR is rich in polar hydrophobic residues and has high proline content, similar to IDRs in other proteins [91]. Together, these data suggest the presence of a flexible linear *N*-terminal domain and a rigid globular *C*-terminal domain in the ORF57 tertiary structure (Figure 3B). The predicted secondary structure in ORF57 appears to be conserved in several other ORF57 homologues, including HSV-1 ICP27, HCMV UL69, and EBV EB2 (Figure 4A). A high level of structural similarities among ORF57 homologues implies strongly that the observed common functions among homologues results from their structures rather than from conservation of their protein sequences [92,93,94].

### 5.2. ORF57 Secondary Structure and Function

#### 5.2.1. Roles of the ORF57 *N*-Terminal IDR in ORF57 Function

Due to their high flexibility in different conformations, IDRs are capable of interacting with multiple ligands [100] through several short linear sequence motifs. The multiple contact points between IDRs and ligands can vary among individual ligands [101]. Because of their high binding capacity, IDRs carry out numerous activities, are often found in multifunctional proteins, and are highly prevalent in viral proteomes [102,103,104]. Consistent with this, the IDR-containing ORF57 *N*-terminal domain mediates the majority of ORF57’s interactions with numerous cellular factors, and thus represents a major interaction interface of ORF57 protein (Table 1) [16,20,31,38,56]. The disordered binding region of KSHV ORF57 seems to be highly conserved among HSV-1 ICP27, HCMV UL69, and EBV EB2 (Figure 4B). The requirement for extensive mutations, such as simultaneous mutation of at least 2 NLSs, to abolish ORF57 binding activities suggests the presence of multiple binding motifs that mediate ORF57 interaction with cellular factors [16]. The ORF57 IDR might represent a monovalent domain capable of binding only one factor at a time. The presence of competitive binding between RBM15 and SRSF3 to ORF57 in the regulation of K8β RNA splicing strongly supports this hypothesis [31]. Competitive binding of cellular factors to ORF57 depends on their expression level and may represent a new mode in the regulation of individual ORF57 activities, which may explain the observed cell-type differences in ORF57 activity. Binding between ORF57 and cellular factors occurs between the IDR of ORF57 and the structured domains of the cellular factors. With an increasing number of mapped interaction motifs in cellular factors, it has become clear that ORF57 prefers to interact with the RRM or other RNA binding motifs of cellular factors, including those of Aly/REF, PCBP1, PABPC1, and SRSF3 (Table 1). Consequently, these interactions with ORF57 often cooperate with or affect these RNA-binding proteins from binding to RNA. For example, ORF57 interaction with PABPC1 alleviates the suppressive effect of PABPC1 on PAN expression [37]. Binding of the ORF57 *N*-terminus to SRSF3 prevents inhibitory SRSF3 from associating with the KSHV K8β intron and thereby promotes K8β splicing (Figure 2) [31]. Similarly, the Aly/REF-RNA interaction can be blocked by a peptide derived from the HVS ORF57 *N*-terminus [41].

**Table 1:** ORF57 cofactors.

ORF57 cofactor	Cofactor function	Interaction region in ORF57 (aa)	Interaction region in cofactor (aa/Domain)	Proved direct interaction	Proposed function of interaction	Conserved interaction in homologues	Citation
**Cellular cofactors**							
**Aly/REF**	RNA-binding protein	1–251 (181–215)	103–163 (RRM)	+	RNA stability/Export (?)	+	[16,36]
**CBP80**	RNA-binding protein	ND	ND	−	RNA stability/Export (?)/Translation	−	[45,56]
**CKII α**	Protein kinase	181–455	ND	+	Phopshorylation	+	[105]
**CKII α'**	Protein kinase	387–455	ND	+	Phopshorylation	+	[105]
**CKII β**	Protein kinase	181–215	150-182	+	Phopshorylation	+	[105]
**hnRNP K**	RNA-binding protein	17–181/329–387	240–337 (KI) interactive domain	+	?	+	[105]
**hnRNP U**	RNA-binding protein	1–251	ND	−	?	−	[90]
**Nup62**	Nucleoporin	ND	ND	−	?	+	[106]
**NXF1**	RNA export factor	ND	ND	−	Export (?)	+	[56]
**OTT3**	RNA-binding protein	1–251	488–890 (SPOC)	+	RNA stability/Polyadenylation	+	[38]
**PABPC1**	RNA-binding protein	1–251	1–370 (RRM1-4)	+	RNA stability	−	[56]
**PCBP1**	RNA-binding protein	179–205	48–96 (KH-I)	+	Translation	−	[74]
**PYM**	Scaffold protein	ND	ND	−	Translation	−	[75]
**RBM15**	RNA-binding protein	1–251	530–977 (SPOC)	+	RNA stability/Polyadenylation	+	[38]
**SRSF1**	RNA-binding protein	ND	ND	−	RNA splicing	+	[20]
**SRSF3**	RNA-binding protein	1–251	1–83 (RRM)	−	RNA splicing	+	[31]
**U snRNPs**	Splicing factors	1–251	ND	−	RNA splicing	+	[20]
**U2AF35**	RNA-binding protein	1–251	ND	−	RNA splicing	+	[20]
**UAP56**	RNA-binding protein	ND	ND	−	Export (?)	+	[45]
**UBE2O**	Ubiqitin-conjugating enzyme E2O	1-251	ND	-	?	-	[90]
**UIF**	RNA-binding protein	ND	ND	+	Export (?)	−	[50]
**Viral cofactors**								
**K-bZIP**	Transcription factor	96–215	ND	+	?	−	[85,86]
**ORF23**	?	ND	ND	+	?	−	[86]
**ORF50**	Transcription factor	ND	1–518 (E3-LR)	+	Transactivation (?)	−	[83,84,86]
**ORF52**	?	ND	ND	+	?	−	[86]
**ORF57**	Posttranscriptional regulator	251–455	251–455	+	Self-interaction	+	[86,90,105]
**ORF61**	Ribonucleotide reductase	ND	ND	+	?	+	[86]
**ORF63**	Tegument	ND	ND	+	?	−	[86]
**ORF67.5**	?	ND	ND	+	?	+	[86]
**ORF68**	Glycoprotein	ND	ND	+	?	+	[86]

Cellular and KSHV (viral) proteins found to interact with ORF57 are summarized in this table. An interaction (+) is considered direct only if it has been proved with a GST pulldown assay of the two purified proteins or by using yeast two-hybrid systems. If a cofactor was found to interact with at least one ORF57 homologue, the interaction was considered being conserved. ND—not determined, RRM—RNA-recognition motif, KI—hnRNP K interaction domain, KH-I—K homology domain type I, SPOC—spen paralogue and orthologue *C*-terminal domain, E3—ubiquitin ligase 3 domain, LR—leucine-rich domain. ?—unknown.

Similar to other homologues in the herpesvirus family, KSHV ORF57 is an RNA-binding protein [20,30,31,37,57,58,107], and its *N*-terminus is essential for its interaction with RNA despite lacking a classical RNA-binding domain [20,30]. The arginine-rich region (ARM) within the ORF57 *N*-terminus overlaps the regions of SR/RS dipeptides and three NLSs and is presumably responsible for its RNA-binding activity. Deletion of the RGG box in this region does not affect the binding of KSHV ORF57 to RNA [30]. The ARM motifs also mediate RNA-binding in ORF57 homologues and other RNA-binding proteins [41,108,109,110,111,112,113]. Although purified ORF57 can bind to any RNA indiscriminately, the addition of cellular nuclear extract provides ORF57 with specificity for its RNA targets [15,16]. This essential role of cellular cofactors in ORF57 specificity is further supported by the observation that loss of the ORF57–cofactor interaction results in dissociation of ORF57 from the target RNA [20,56,58]. The finding that ORF57 does not bind to PAN RNA in PABPC1-depleted cell extracts strengthens the observation that ORF57 and PABPC1 bind cooperatively to PAN RNA [56]. HVS ORF57 and Aly/REF also bind cooperatively to RNA [110].

The RNA motif for ORF57-specific binding is not sequence specific, but rather structure dependent [37,56,57,58,66]. Several studies indicate that the secondary structure in a targeted RNA is important for ORF57 interaction. Among 11 viral RNA transcripts specific for ORF57 binding in an ORF57-CLIP assay [58], the identified MRE motifs in viral PAN, vIL-6, and ORF59 RNA contain a hairpin-loop structure with a NGGA loop. Introduction of point mutations into the RNA hairpin-NGGA loop prevents both ORF57 and its cofactor from binding and make the targeted RNA unresponsive to ORF57 [37,56,57,58,66], indicating the importance of the structural motif in ORF57 function.

#### 5.2.2. Phosphorylation of the ORF57 *N*-Terminal Domain

In general, IDRs contain many sites that are preferred targets for posttranslational modification of proteins [97]. Consistent with this, multiple phosphorylated serine/threonine residues are located within the IDRs of KSHV ORF57, HSV-1 ICP27, HCMV UL69, and EBV EB2 (Figure 4C). These phosphorylated residues within the ORF57 IDR can be divided into two groups. S21, S43, and T32 form a group of residues phosphorylated by casein kinase II (CKII) [90] by direct interaction with ORF57 [105]. These three CKII phosphorylation sites are located within the acidic region of ORF57, and their phosphorylation would increase the overall charge in the acidic region. CKII phosphorylation sites have also been mapped in the *N*-termini of the HSV-1 ICP27 and EBV EB2 proteins (Figure 4C), and these sites must be phosphorylated for the proteins to function during viral infection [98,99]. S95 and S97 form the other group identified in proteomic analyses of ORF57 protein [90]. Our studies predict that their phosphorylation is mediated by GSK3 (glycogen synthase kinase 3); however, S95 could also be a substrate of p38 MAPK (mitogen-activated protein kinase p38) and S97 could be a substrate of RSK (ribosomal S6 kinase) [90].

#### 5.2.3. Homodimerization of ORF57 via Its *C*-Terminus

In contrast to the *N*-terminus, the ORF57 *C*-terminus represents a highly structured rigid domain enriched in α-helixes (Figure 3). Due to the existence of relatively higher sequence conservation among the homologues, the *C*-terminus was initially considered to be a major functional domain of ORF57 [94,114], but this was later ruled out because deletion of the majority of the ORF57 *C*-terminus (aa 252–455) does not significantly affect ORF57 activity [16,20,56,58]. As the structured protein regions are less tolerant to mutation than the unstructured parts, the presence of a relatively higher sequence homology among the homologues’ *C*-termini could simply reflect the conservation of protein structure rather than function.

The *C*-terminus of ORF57 mediates ORF57 self-interaction, a feature that has been found in several homologues of the ICP27 family [92,94,105,115]. ORF57 forms homodimers and even homotrimers *in vitro* and homodimers *in vivo* via its *C*-terminus [90]. By screening a library of small peptides derived from the ORF57 *C*-terminus, we have recently identified the *C*-terminal α-helixes 7–9 as being responsible for ORF57 homodimerization. Introduction of point mutations in α-helix 7 (aa 280–299) prevents ORF57 from homodimerizing. Interestingly, these regions exhibit high conservation in both protein structure and amino acid sequence among ORF57 homologues [90].

## 6. Regulation of ORF57 Stability by Phosphorylation and Homodimerization

**Figure 5:**
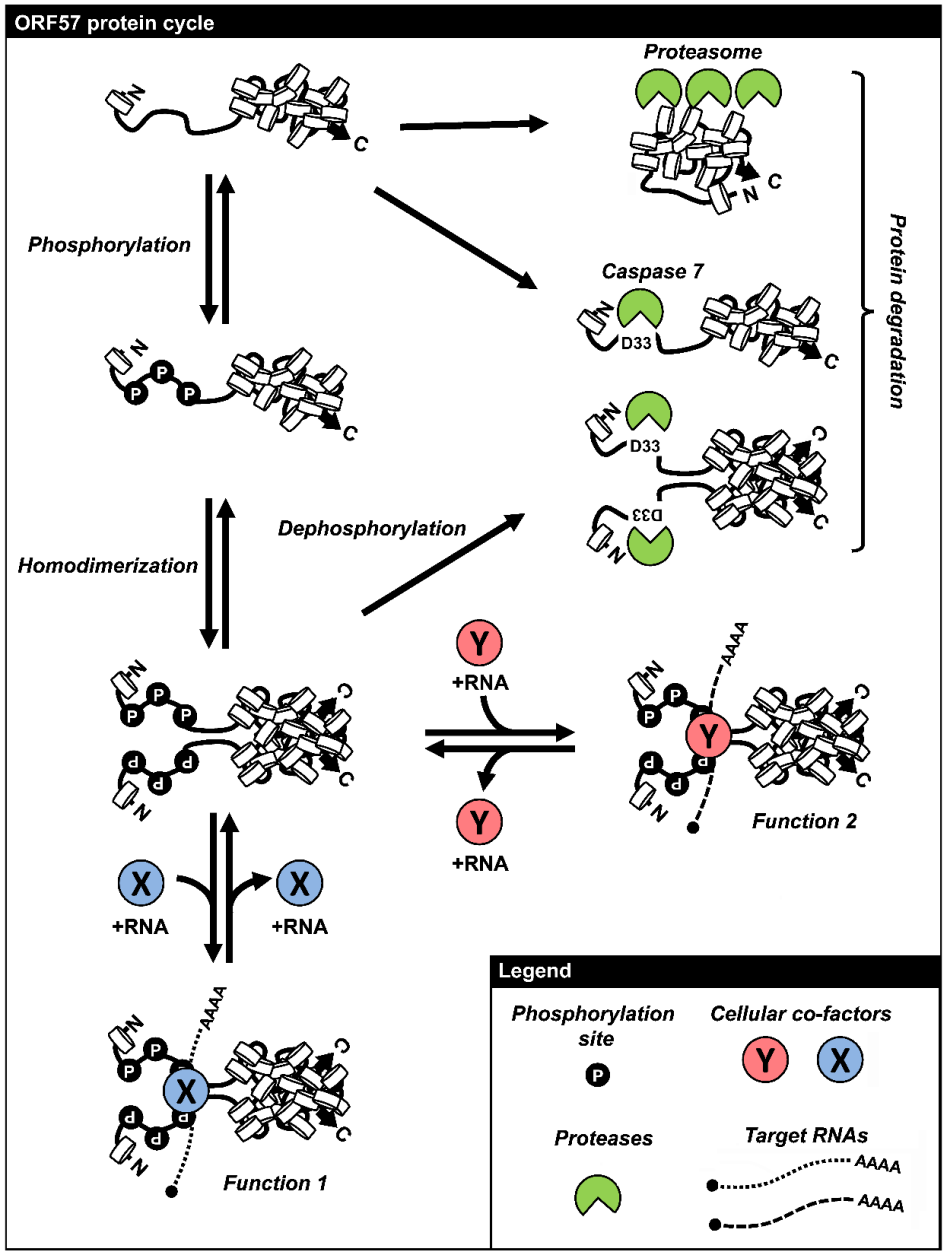
The life cycle of ORF57 protein. KSHV ORF57 features an intrinstically disordered *N*-terminal domain and a highly structured *C*-terminal domain. This protein is translated initially as a monomeric protein and undergoes the protein *N*-terminal phosphorylation by host CKII or other kinases. The monomeric form of ORF57 subjects to cleavage by caspase 7 or degradation via proteasomal pathway. Two phosphorylated monomeric ORF57 polypeptides dimerize through the *C*-terminal α-helixes 7–9, resulting in the formation of an ORF57 homodimer. ORF57 homodimers interact with cellular protein-RNA complexes to exert various functions of ORF57 and otherwise may subject to caspase 7 digestion. This figure is reproduced with permission from [90].

Due to the high level of ORF57 expression during KSHV lytic infection, the stability of ORF57 protein was not previously considered as a regulatory mechanism of ORF57 activity. We have recently uncovered two modes of regulation of ORF57 protein stability and linked each mode to an individual ORF57 domain (Figure 5). The first mode is caspase-mediated cleavage of ORF57 and production of a truncated ORF57 protein during KSHV lytic infection [116]. ORF57 cleavage is mediated primarily by the effector caspase 7, which is activated in response to virus reactivation. The cleavage occurs at aspartic acid residue D33 within the *N*-terminal acidic region of ORF57 (Figure 1). In KSHV-infected cells, ORF57 cleavage predominantly occurs in the cytoplasm enriched in activated caspase 7, and the cleavage correlates with the amount of activated caspase 7. The cleaved ORF57 protein displays reduced activity for regulating ORF56 and ORF59 expression and K8 RNA splicing. Conversely, inhibition of caspase activity or knockdown of caspase 7 expression in KSHV-infected cells protects ORF57 from caspase cleavage and promotes the higher expression of viral lytic genes, ultimately increasing the production of virions [116]. Thus, caspase 7 cleavage of ORF57 acts as a cellular defense mechanism against KSHV infection. However, the susceptibility of ORF57 to caspase 7 cleavage is preventable by CKII phosphorylation of the T32 within the caspase 7 recognition motif 30-DETD-33. Dephosphorylation of ORF57 protein accelerates its cleavage by caspase 7, whereas ORF57 becomes resistant to caspase 7 cleavage after *in vitro* phosphorylation with CKII [90]. Other studies also show that phosphorylation of serines or threonines in close proximity to or within a caspase cleavage site affects the cleavage of caspase substrates [117,118]. Thus, the regulation of ORF57 caspase cleavage by CKII provides an important link between CKII activity and productive KSHV infection, consistent with CKII’s anti-apoptotic effect and activation of CKII activity and its relocalization to the cytoplasm by ICP27 during HSV-1 infection [119].

Mutation of the conserved residues in the identified dimer interface inhibits ORF57 homodimerization, but induces rapid degradation of monomeric ORF57 mutant protein. A recent study showed that the ORF57 homodimer has a half-life of more than 4 h, but the half-life of its monomeric mutant form is about only ~1 h [90]. However, the degradation of monomeric ORF57 mutant appears to be induced by a proteasome-mediated pathway and thus could be blocked by MG132, a proteasomal inhibitor. Accumulation of the mutant ORF57 in a high-molecular-weight form can occur in the presence of MG132 and is resulted from polyubiquitination of the mutant ORF57. The expression of wt ORF57 protein does not respond to inhibition by MG132 and displays no high-molecular-weight form [90]. Thus, dimerization allows the ORF57 protein to escape from proteasome-mediated degradation and is critical for rapid accumulation of ORF57 for productive KSHV infection.

## 7. Remarks and Perspectives

Work by several research groups over the past decade has enormously extended our understanding of KSHV ORF57 function and its mechanisms of action. KSHV ORF57 is a truly multifunctional protein and affects RNA biogenesis at various lytic infection stages (Table 2). The three major defined functions of KSHV ORF57 during KSHV lytic infection are the enhancement of RNA stability, promotion of alternative RNA splicing, and control of protein translation (Figure 2). KSHV ORF57 does not function as a bona fide export factor. Despite being characterized as a member of the HSV-1 ICP27 family, KSHV ORF57 deviates from the prototype ICP27 protein in several ways. For intronless viral RNA, the main function of ORF57 is stabilizing the RNA, whereas for ICP27 it is exporting the RNA. For intron-containing viral RNA, KSHV ORF57 functions as a viral splicing factor to promote splicing of the viral RNA, whereas ICP27 functions as an inhibitor of RNA splicing. However, EBV EB2, a close homologue of KSHV ORF57, resembles KSHV ORF57 in this regard and activates RNA splicing. These data clearly indicate that the functional evolution of individual homologues contributes to the unique lytic life cycles and pathogenesis of the different members of the herpesvirus family.

Our understanding of the molecular mechanism underlying ORF57 function has advanced in several new directions. The multifunctionality of ORF57 is attributed to its ability to interact with numerous cellular proteins. Identifying the full interactome of ORF57 would hold the promise of discovering new, not-yet-described functions of ORF57. The finding that ORF57 can affect the expression of hIL-6 and splicing of other non-KSHV RNAs with a suboptimal intron strongly indicates its potential impact on the host transcriptome, which has not yet been determined. In particular, we may shift our attention in the coming years to exploring the role of KSHV ORF57 in the biogenesis of the fast-growing class of host non-coding regulatory RNAs.

It is worth noting that KSHV ORF57 does show nonspecific effects on almost all examined transgenes of both viral and non-viral origin. This nonspecific effect increases gene expression, independently of the promoter, sequence composition, length, and coding potentials of the target genes. The mechanism of this nonspecific effect of ORF57 is not fully understood and could be posttranscriptional. Therefore, any future description of a novel ORF57 function must be clearly distinguishable from this nonspecific effect. Second, the binding of ORF57 to cellular cofactors often changes the properties of these cofactors, including the subcellular localization, binding properties, and ultimately their functions. Thus, the functional consequences of the identified interactions would not be a simple extrapolation of the existing function(s) of the ORF57-interacting protein(s). Third, mutation and truncation experiments are important tools for dissecting the sequence motifs involved in ORF57 functions, but mutation or truncation may change the protein folding and thereby expose other motif(s) that are covered during normal folding. This could affect the activities of ORF57 by changing its interactions with other cellular proteins. Thus, future studies to correlate ORF57 protein structure, folding, and function are urgently needed in order to understand the biochemical and biophysical properties of individual ORF57 protein domains and their contribution to each of the ORF57 functions.

**Table 2:** Transcripts regulated by ORF57.

*ORF57 RNA target*	Expression stage	Coding potential	Identified MRE element	ORF57 activity toward the target	Citation
Viral RNA targets					
*vIL6 (K2)*	E	Cytokine	MRE A/B	RNA stability/translation	[58]
*K4*^#^	E	Chemokine	ND	?	[58]
*K8*	E	Transcription factor (k-bZIP)	K8 intron 2	Splicing	[20,31]
*K8.1*^#^	L	Glycoprotein	ND	?	[8]
*K9*^#^	E	vIRF-1	ND	?	[58]
*K12*^#^	L	Kaposin	ND	?	[58]
PAN	E	lncRNA	MRE I–III	RNA stability	[8,37,39,55]
*mPC*^#^	L	Minor capsid protein	ND	?	[9]
*ORF4*	E	Complement regulatory protein	ND	RNA stability (?)	[10]
*ORF6*	E	Single-stranded DNA binding protein	ND	RNA stability (?)	[10]
*ORF7*^#^	L	Terminase subunit	ND	RNA stability (?)	[10]
*ORF8*^#^	L	Glycoprotein B	ND	RNA stability (?)	[10]
*ORF9*^#^	E	DNA polymerase	ND	?	[9,10]
*ORF17*^#^	L	Viral protease	ND	?	[58]
*ORF47*	L	Glycoprotein	ND	RNA stability	[40,45]
*ORF50*	IE	Transcription factor (Rta)	ND	Splicing	[20,58]
*ORF52*^#^	L	Tegument protein	ND	?	[58]
*ORF56*	E	Primase	ND	RNA stability	[22,40]
*ORF59*	E	DNA polymerase processing factor	5' MRE	RNA stability	[10,16,55,66]
*ORF60*^#^	E	Ribonucleotide reductase subunit	ND	RNA stability (?)	[10]
*ORF61*^#^	E	Ribonucleotide reductase subunit	ND	RNA stability (?)	[10]
*ORF73*^#^	L	LANA	ND	?	[58]
*Non-viral RNA targets*					
*hIL6*	N/A	Cytokine	miR-608 binding site	Translation	[58]
*BPV-1 L1/L2*	N/A	Capsid proteins	SE4	Splicing	[31]
*HPV16 L1/L2*	N/A	Capsid proteins	ESE	Splicing	[31]

The targets marked with # are downregulated in cells infected with an ORF57-null KSHV virus or associated with ORF57 protein in JSC-1 cells by anti-ORF57 CLIP, but their response to ORF57 has not yet been proven with a co-transfection assay. BPV-1—bovine papilloma virus 1, HPV16—human papillomavirus type 16, IE—immediate early, E—early, L—late, SE4—splicing enhancer 4, ESE–exonic splicing enhancer, N/A—not applicable, ND—not determined, MRE—Mta-responsive element.
